# 6p22.3 deletion: report of a patient with autism, severe intellectual disability and electroencephalographic anomalies

**DOI:** 10.1186/1755-8166-6-4

**Published:** 2013-01-17

**Authors:** Daniela Di Benedetto, Giuseppa Di Vita, Corrado Romano, Mariangela Lo Giudice, Girolamo Aurelio Vitello, Marinella Zingale, Lucia Grillo, Lucia Castiglia, Sebastiano Antonino Musumeci, Marco Fichera

**Affiliations:** 1 Laboratory of Medical Genetics, I.R.C.C.S. Associazione Oasi Maria Santissima, Troina, Italy; 2 Unit of Pediatrics and Medical Genetics, I.R.C.C.S. Associazione Oasi Maria Santissima, Troina, Italy; 3 Unit of Neuromuscular Disease, I.R.C.C.S. Associazione Oasi Maria Santissima, Troina, Italy; 4 Unit of Neurology, I.R.C.C.S. Associazione Oasi Maria Santissima, Troina, Italy; 5 Unit of Psychology, I.R.C.C.S. Associazione Oasi Maria Santissima, Troina, Italy; 6 Medical Genetics, University of Catania, Catania, Italy

**Keywords:** 6p22.3 deletion, Array-CGH, ASDs, Hypotonia, DTNBP1

## Abstract

**Background:**

The interstitial 6p deletions, involving the 6p22-p24 chromosomal region, are rare events characterized by variable phenotypes and no clear genotype-phenotype correlation has been established so far.

**Results:**

High resolution array-CGH identified 1 Mb *de novo* interstitial deletion in 6p22.3 chromosomal region in a patient affected by severe Intellectual Disability (ID), Autism Spectrum Disorders (ASDs), and electroencephalographic anomalies. This deletion includes *ATXN1*, *DTNBP1*, *JARID2* and *MYLIP* genes, known to play an important role in the brain, and the *GMPR* gene whose function in the nervous system is unknown.

**Conclusions:**

We support the suggestion that *ATXN1*, *DTNBP1*, *JARID2* and *MYLIP* are candidate genes for the pathophysiology of ASDs and ID, and we propose that deletion of *DTNBP1* and/or *JARID2* contributes to the hypotonia phenotype.

## Background

The interstitial deletions involving the 6p22-p24 chromosomal region are characterized by variable phenotype, according to the different size of the deleted regions and the small amount of patients reported to date. The clinical phenotype includes psychomotor delay, hypotonia, defects in brain, heart, kidney and eye, short neck, craniofacial malformations, as well as clinodactyly or syndactyly [1-4]. Recently, six patients carrying overlapping deletions in the 6p22.3-p24.3 region were described. They presented common features of 6p deletion syndrome, but also neurological or behavioral abnormalities, including speech delay, autism spectrum disorders (ASDs), hyperactivity/ADHD, or other behavioral abnormalities [5]. Here, we report a 1 Mb interstitial deletion in the 6p22.3 chromosomal region identified by 60-mer oligonucleotide 180K array comparative genomic hybridization (array-CGH) in an 18 years-old patient affected by severe ID, ASDs and electroencephalographic (EEG) anomalies. Further analysis demonstrated that the deletion was *de novo* and microsatellite segregation analysis revealed that the rearrangement involved the paternal chromosome 6. Four genes (*ATXN1*, *DTNBP1*, *JARID2* and *MYLIP* ) located in the deleted 6p22.3 region are involved in the development and function of the nervous system [4]. Our finding supports the contributory effect of these genes in the pathophysiology of ASDs and ID [4,5]. Furthermore, we propose a possible involvement of *DTNBP1* and/or *JARID2* genes in hypotonia.

### Clinical description

The patient is the first of two siblings born to healthy non-consanguineous parents. The pregnancy was characterized by weak fetal movements and he was born at term by induced vaginal delivery. His birth weight was 4250 g (> 97^th ^centile) and he showed physiological jaundice, treated by phototherapy. Dacryocystitis by lacrimal duct stenosis, bilateral hydrocele, umbilical hernia and left lateral xiphoid epigastric hernia were observed.

The patient had delayed psychomotor development, he walked at 15 months and later he showed autonomous deambulations with awkward movements. He presented with delayed speech. We first observed the patient at the age of 3 years and 9 months. The patient presented with long face, drooling, epicanthic folds, macrosomia (head circumference, height and weight > 97^th ^centile), central obesity, large testes, knock knees and bilateral valgus flat foot. Diffuse ligament laxity and muscular hypotonia, but with standard tropism and no deficit of strength, where also present. When observed at the age of 12 years and 7 months, the patient showed normal deep tendon reflexes, shifting with age to hyporeflexia, especially at lower limbs. He had poor fine motor skills and general coordination. His gait was broad-based with clampsy movements.

The neurological examination, performed at the age of 3 years and 9 months, showed severe ID and typical autistic traits, impairments in environmental and social interactions, avoidance of spontaneous or requested eye contact, very limited verbal and non-verbal communication, limited interests, and repetitive behaviors with frequent hand flapping, head and trunk rocking. This behavioral phenotype was classified in the autism spectrum according to ADOS-G and ADI-R testing. The patient was clearly hyperactive and presented with behavioral problems including self-injury (head banging) and, less frequently, aggressiveness (biting) towards others. He suffered from sleep disorders.

Magnetic resonance (MR) imaging performed at the age of 4 years and 6 months revealed mild abnormalities in the posterior periventricular white matter and enlarged sulci and ventricles. EEG showed paroxysmal discharges, such as spikes and spike-and-wave complexes, isolated or in short sequences that occurred in the central and temporal regions of both hemispheres, significantly activated during sleep.

Electromyography (EMG) showed an increased number of polyphasic low-amplitude potentials (the patient did not cooperate for electroneurography).

Heart and kidney ultrasounds were normal (asymptomatic kidney microlithiasis). Metabolic screening and fragile X testing were normal. Conventional cytogenetic studies showed a normal male karyotype (46,XY).

## Results

**Figure 1:**
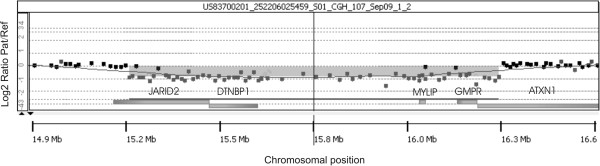
**Array**-**CGH deletion.** Detailed view of 6p22.3 segment with in the Y-axis the log2 signal ratio of chromosome 6 probes (patient /reference) plotted as a function of chromosomal position (X-axis). The shaded region represents the 1 Mb deletion identified in our patient.

We have identified in our patient a *de novo* interstitial deletion of 1 Mb in the 6p22.3 chromosomal region as described in Figure 1. No other significant CNV was detected. The deletion involved five genes *ATXN1*, *DTNBP1*, *JARID2*, *MYLIP* and *GMPR*. The distal breakpoint was located in intron 1 of *JARID2* gene, between genomic position 15.28 and 15.29 Mb, while the proximal breakpoint was located in intron 7 of *ATXN1* gene, between the genomic position 16.36 and 16.37 Mb (according to human genome assembly, February 2009, hg19, http://genome.ucsc.edu/). Minimal and maximal sizes of deletion were 1.07 Mb and 1.09 Mb, respectively. This aberration was not reported in the online Database of Genomic Variants, a curated collection of structural variations identified in healthy control samples (http://projects.tcag.ca/variation/?source=hg19), and was not present in the parental samples investigated by array-CGH. Further microsatellite segregation analysis with markers located in the deleted segment, confirmed the deletion and demonstrated a paternal origin of this rearrangement (data not shown).

## Discussion

**Figure 2:**
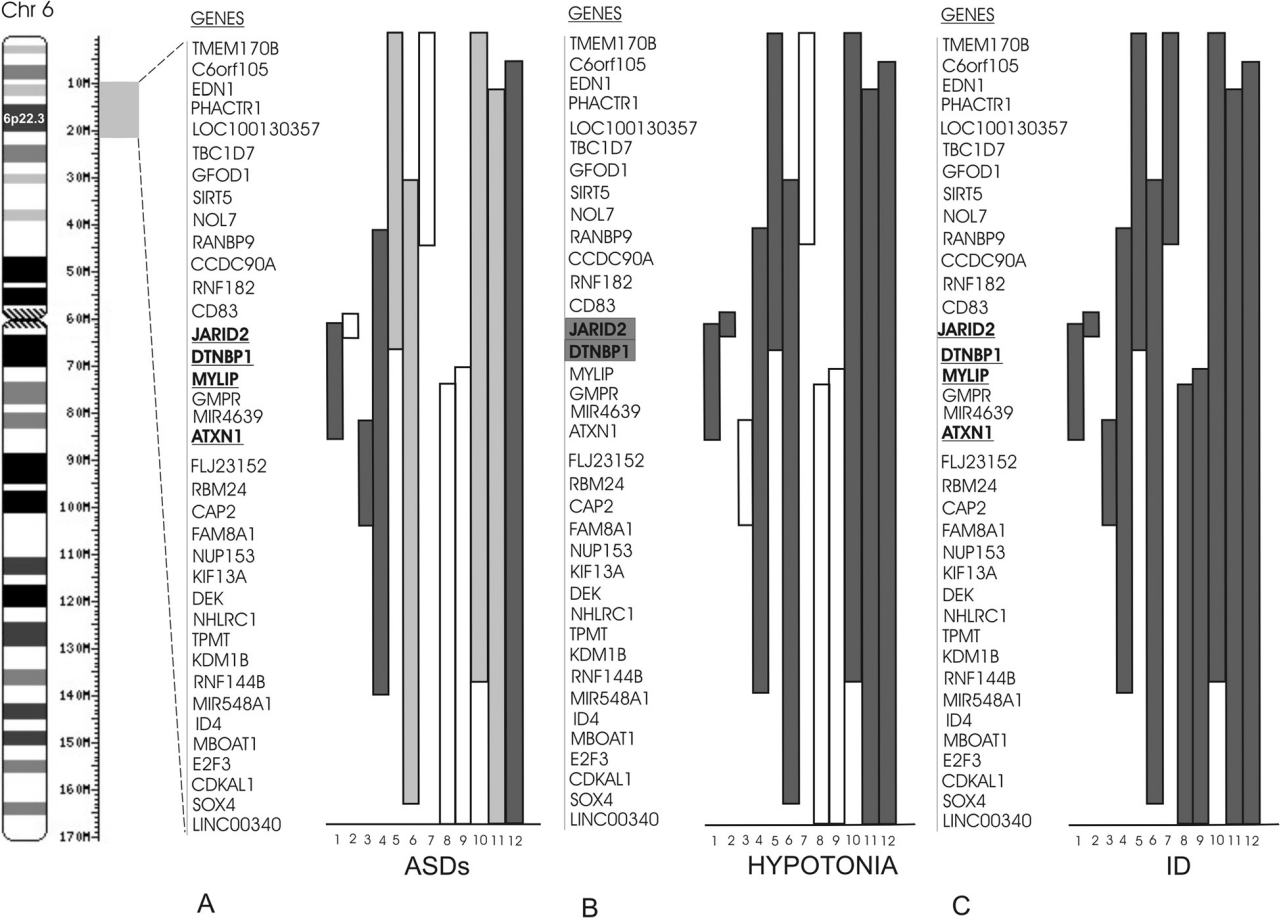
**Representation of genotype**-**phenotype correlations of 6p22**.**3 deletions.** Schematic representation of genotype-phenotype correlations in patients with 6p22.3 deletions on 3 physical maps named “ASD” (**A**), “Hypotonia” (**B**) and “ID” (**C**). The grey block represents 10Mb of 6p chromosomal region; genes in the 6p22.3 genomic interval are represented (not in scale) with candidate genes for ID and ASDs in bold, underlined, and candidate genes for hypotonia in grey blocks. The numbered columns (1–12) represent 12 deletions in 12 patients. For the concerned features, dark bars represent symptomatic patients, white bars represent asymptomatic patients, and light grey bars represent unassessed patients. 1: Present case; 2: Zvi et al., 2011 [6]; 3: Patient 2 [5] (no formal ASDs testing was performed); 4: Patient 1 [5]; 5: Patient 95–800 [1]; 6: Patient P1 [2]; 7: Patient 4 [5]; 8: Patient 5 [5]; 9: Bremer et al., 2009 [4]; 10: Patient P2 [2]; 11: Patient 91–145 [1]; 12: Patient 6 [5] (Although a formal ASDs testing was not performed, this patient showed ASDs associated features such as hyperactivity, speech delay, behavioral abnormalies).

The interstitial deletions of 6p are characterized by variable sizes and phenotypes. A minimal critical region (MCR) for the 6p22 deletion phenotype has been proposed by Bremer et al. [4] who identified a 2.2 Mb region involving 12 genes. Some of them were proposed as candidate genes, but none has been strongly associated with specific clinical features of the syndrome. Here we report a 6p deletion of about 1 Mb encompassing five genes (*ATXN1*, *DTNBP1*, *JARID2*, *MYLIP* and *GMPR*), identified in a patient with severe ID, hypotonia, brain anomalies, EEG abnormalities, macrosomia, obesity, and ASDs with associated hyperactivity and behavioral abnormalities. In Figure 2 we present a genotype-phenotype correlation between our patient and others reported in the literature [1-6] and we observe that the phenotype of our patient overlaps with previously reported cases of 6p22.3 deletions, particularly for ID, ASDs and hypotonia. Our findings support the suggestion [4,5], that *ATXN1*, *DTNBP1*, *JARID2* and *MYLIP* are candidate genes for ID and ASDs. Furthermore, we suggest that two deleted genes (*JARID2* and/or *DTNBP1*) can be involved in hypotonia.

The deletion of *ATXN1* gene could be associated to ID and ASDs. The ataxin-1 protein is localized in the neurons of the basal ganglia, pons, cortex and Purkinje cells of the cerebellum and it is involved in the regulation of transcription and RNA processing. *ATXN1* is the cause of spino-cerebellar ataxia type 1 (SCA1, OMIM:164400), a dominantly inherited neurodegenerative disease, through CAG repeat expansion with a gain-of-function mechanism [7], while loss-of-function of *ATXN1* seems to lead to motor coordination impairments and learning deficits [8]. Researchers have found a significant association between SNPs in the gene and intelligence quotient in ADHD patients [9] and also a correlation among loss-of-function of *ATXN1* and increased amyloid β-protein levels in Alzheimer patients [10].

*JARID2* gene is a DNA-binding transcriptional repressor. It is expressed in embryonic and adult human neurons and it is a critical factor for the cardiovascular development [11,12]. Furthermore, a significant association between a SNP (rs7766973) in the *JARID2* gene and autism has been reported [13].

The myosin regulatory light-chain interacting protein (*MYLIP*) is a novel member of the ezrin-radixin-moesin protein family (ERM). These proteins participate in the regulation of cell proliferation, differentiation, and signals transduction [14]. Olsson et al. [15] found that MYLYP is expressed in rat embryonic hippocampal neuronal cell bodies and growth cones, where it works as a neurite outgrowth suppressor [16]. MYLIP is also known as IDOL, inducible degrader of the LDL receptor, based on its involvement in cholesterol regulation [17]. IDOL knockdown in hepatocytes increases LDLR protein levels and promotes LDL uptake through LXR-IDOL-LDLR pathway [18]. The impairment of this function can be related to the obesity observed in our patient.

*DTNBP1* is a gene with several important functions. The encoded protein interacts with α- and β-dystrobrevins as component of the dystrophin protein complex (DPC) in muscle and brain, mediating the link between the DPC and intracellular signaling cascade. In muscle, it functions as a structural scaffold linking the α-dystrobrevins to sarcolemma [19] and mutations which disrupt the DPC can give rise to numerous types of skeletal myopathy, including Duchenne muscular dystrophy (DMD [MIM 310200]) in humans and mice [20]. In the mouse brain, dysbindin is enriched in regions implicated in synaptic structure and signaling of the glutamatergic neuronal system [19]. Numakawa et al. [21] suggest that dysbindin might influence exocytotic glutamate release via upregulation of the molecules in pre-synaptic machinery as synaptosomal-associated protein (*SNAP25*) and synapsin 1 (*SYN1*). These results suggest that dysbindin might be one of the regulatory proteins in the excitatory neurotransmission and can be associated also with EEG anomalies observed in our patient.

Moreover, *DTNBP1* has been connected with autistic features observed sometimes in patients with Duchenne muscular dystrophy. Hendriksen et al. [22] reported in a population of males with Duchenne muscular dystrophy a neuropsychiatric comorbidity, including increased frequencies of ASDs and ADHD.

The Guanosine monophosphate reductase (GMPR) catalyzes the irreversible NADPH-dependent reductive deamination of guanosine monophosphate (GMP) to inosine monophosphate (IMP) and is involved in thermogenesis [23], but its function in the nervous system is unknown.

## Conclusions

Using array-CGH, we identified 1 Mb deletion in 6p22.3 in a male patient with ASDs, ID, and EEG anomalies and we delineate a genotype-phenotype correlation for 6p22.3 deleted patients. We suggest that *JARID2* and/or *DTNBP1* genes can be candidate for hypotonia and we support the role of *ATXN1*, *DTNBP1*, *JARID2* and *MYLIP* genes in the pathophysiology of ASDs and ID [4,5]. Incomplete penetrance of ASDs, as shown in Figure 2A, may be due to other genetic, epigenetic or environmental factors.

Additional clinically and molecularly well-defined, patients will be required to improve the genotype-phenotype correlation of the 6p22-p24 deletion syndrome.

## Methods

### Consents

The study was approved by the local ethics committee on 18 May 2012 and written informed consent was obtained for the patient for publication of this report.

Genomic DNAs were extracted by standard procedures, and tested for submicroscopic copy number variants using the commercially available Human Genome array-CGH 180K Microarray (Agilent Technologies, Palo Alto, CA), which provides an average resolution of around 13 Kb. The patient DNA was matched versus a male reference DNA (NA10851, Coriell Institute), and the assay performed according to the manufacturer’s protocol version 7.1. Arrays were scanned using the Agilent Microarray Scanner. Data were extracted using Feature Extraction Software version 10.7 and analyzed using Agilent Genomic Workbench Lite Edition 6.5. The parental samples were investigated with 180K array-CGH platform, and parental origin was tested with microsatellite analysis of an existing polymorphic marker (D6S260) and two new markers developed in this study (JAR1, forward primer 5^′^-GCCTTCTTTAACCGCTTGCA-3^′^, reverse primer 5^′^-CCTCATGGTTACTGCATCCTGTAA-3^′^, and JAR2 forward primer 5^′^-CTGCTATGGATTCATGGCCG-3^′^, reverse primer 5^′^-CCTGCCTTCCAAATGTGTTATACA-3^′^) all located inside the JARID2 gene.

The PCR products were electrophoresed on a ABI 310 Genetic Analyser (Applied Biosystems) and analyzed by Genescan software.

## Abbreviations

ID: Intellectual disability; ASDs: Autism spectrum disorders; ADHD: Attention deficit hyperactive disorders; EEG: Electroencephalography; MRI: Magnetic resonance imaging; EMG: Electromyography; CGH: Comparative genomic hybridization; MCR: Minimal critical region; DPC: Dystrophin protein complex; ERM: Ezrin-radixin-moesin protein family; DMD: Duchenne muscular dystrophy.

## Competing interests

The authors declare that they have no competing interests.

## Authors’ contributions

DDB and PDV designed the experiment and drafted the manuscript. LG, LC and DDB performed the chromosomal microarray assay and data analysis. MLG performed the microsatellite analysis. MZ performed the ADOS-G and ADI-R tests. CR, PDV and SAM examined clinically the patient. GAV performed the EMG analysis. MF drafted the manuscript. All authors read and approved the final manuscript.

## Aknowledgments

This study was supported by Telethon (grant no. GGP08226 to M.F.).
